# Myeloid-Derived Suppressor Cells (MDSC) in Melanoma Patients Treated with Anti-PD-1 Immunotherapy

**DOI:** 10.3390/cells12050789

**Published:** 2023-03-02

**Authors:** Katarzyna Tomela, Bernadeta Pietrzak, Łukasz Galus, Jacek Mackiewicz, Marcin Schmidt, Andrzej Adam Mackiewicz, Mariusz Kaczmarek

**Affiliations:** 1 Department of Cancer Immunology, Poznan University of Medical Sciences, 61-866 Poznan, Poland; 2 Doctoral School, Poznan University of Medical Sciences, 60-812 Poznan, Poland; 3 Department of Food Biotechnology and Microbiology, Poznan University of Life Sciences, 60-627 Poznan, Poland; 4 Department of Medical and Experimental Oncology, Institute of Oncology, University of Medical Sciences, 60-355 Poznan, Poland; 5 Department of Diagnostics and Cancer Immunology, Greater Poland Cancer Centre, 61-866 Poznan, Poland

**Keywords:** melanoma, immunotherapy, anty-PD-1, MDSC

## Abstract

Myeloid-derived suppressor cells (MDSC) are a subset of immature myeloid cells with suppressive activity well described in the context of cancer. They inhibit anti-tumour immunity, promote metastasis formation and can lead to immune therapy resistance. In a retrospective study, blood probes of 46 advanced melanoma patients were analysed before the first administration of anti-PD-1 immunotherapy and in the third month of treatment for MDSC, immature monocytic (ImMC), monocytic MDSC (MoMDSC) and granulocytic MDSC (GrMDSC) by multi-channel flow cytometry. Cell frequencies were correlated with response to immunotherapy, progression-free survival (PFS) and lactate dehydrogenase (LDH) serum level. Responders to anti-PD-1 therapy had higher MoMDSC levels (4.1 ± 1.2%) compared to non-responders (3.0 ± 1.2%) (*p* = 0.0333) before the first administration of anti-PD-1. No significant changes in MDSCs frequencies were observed in the groups of patients before and in the third month of therapy. The cut-off values of MDSCs, MoMDSCs, GrMDSCs and ImMCs for favourable 2- and 3-year PFS were established. Elevated LDH level is a negative prognostic factor of response to the treatment and is related to an elevated ratio of GrMDSCs and ImMCs level compared to patients’ LDH level below the cut-off. Our data may provide a new perspective for more careful consideration of MDSCs, and specially MoMDSCs, as a tool for monitoring the immune status of melanoma patients. Changes in MDSC levels may have a potential prognostic value, however a correlation with other parameters must be established.

## 1. Introduction

Myeloid-derived suppressor cells (MDSC) are described as a heterogeneous population of myeloid cells, immature states that originate from the bone marrow. Although the main characterization of these cells was strongly associated with pathological conditions such as cancer, autoimmune disease and infections due to their strong ability to suppress T-cells and other immune populations, MDSCs are also involved in non-pathological settings such as pregnancy. Their regulatory capacity plays an important role in almost any process in which the immune system is involved [1,2,3,4,5].

Increased frequency of MDSC is observed in many cancer types, including renal cell carcinoma, non-small cell lung carcinoma (NSCLC), prostate cancer or melanoma [6,7,8,9]. The villainous role of MDSC in cancer development results from immune suppression, tumour angiogenesis, drug resistance, and promotion of tumour metastases [10]. Tumour metastasis is a process in which two main steps can be highlighted. Physical migration of cancer cells from the primary tumour to the distant organ is the first step, after that, cancer cell colonization of the organ begins and develops into metastases. The mechanisms by which MDSCs establish the pre-metastatic microenvironment in distant organs are mostly unknown, however, there is evidence that MDSCs play an essential role in metastasis formation [11].

Otvos et al. in their study presented CSCs selectively MDSC-mediated immune suppression. In cytokine analysis, they revealed secretion by CSCs of multiple factors that promoted this process, including macrophage migration inhibitory factor (MIF), which was produced at high levels by CSCs [12]. Three years after, Tanriover and Aytac reviewed a hypothesis about the bilateral effects of cancer stem cells (CSCs) and MDSCs in cancer development [13].

MDSCs due to their heterogeneity can be divided into two main subtypes: monocytic (Mo/M-MDSCs) and granulocytic (Gr/PMN-MDSCs). However, there is another, third phenotype of MDSCs that lacks granulocyte or monocyte markers and so is considered as early (E-MDSCs) or immature monocytic cells (ImMC) [14]. The immunosuppressive potential of particular cells was established by Nagatani et al. in an in vitro assay. Isolated MoMDSCs as well as GrMDSC released Interleukin 1RA (IL-1RA), and arginase and suppressed T-cell activation, in the presence of tumour cells. In contrast, early MDSCs did not show this immunosuppressive effect suggesting the different properties of these cells [15].

The mechanisms of immune cell suppression are different in Mo and GrMDSCs; this is due to the production and secretion of different substances—nitric oxide synthase 2 (NOS2) by MoMDSCs and reactive oxygen species (ROS) by GrMDSCs. Moreover, suppression of T-cell proliferation is mediated by arginase 1 (ARG1) secretion which causes inhibition of expression of one of the CD3 chains leading to T-cell apoptosis [16,17]. MDSCs produce cytokines such as transforming growth factor beta (TGF-β) or interleukin 10 (IL-10), prostaglandin E2 (PGE2) and secrete exosomes, all that results in T-cell and NK cell dysfunction but also Treg induction [16,18,19,20]. MDSC can also encourage the spreading of cancer cells through angiogenesis promotion, epithelial–mesenchymal transition (EMT) and mesenchymal–epithelial transition (MET) transition or secretion of tumorigenic factors such as TGFβ, hepatocyte growth factor (HGF) and IL-6 [21].

MDSCs are not only linked with cancer progression but also discussed as a factor of development of resistance to immunotherapy [22]. Due to this fact, it is important to monitor changes in cell frequencies in order to recognize the occurrence of secondary resistance to therapy as soon as possible.

Melanoma is one of the most fatal types of skin cancer. The number of people diagnosed with and dying from invasive melanoma is growing year by year and the highest mortality rate is referred to patients diagnosed at an advanced stage of the disease [23,24]. In the last decade, the treatment of metastatic melanoma has improved significantly due to the application of immune checkpoint inhibitors (ICI). The mechanism of those agents is based on blocking co-inhibitory T-cells receptors: cytotoxic T-lymphocyte-associated protein 4 (CTLA-4) or programmed cell death protein 1 (PD-1), respectively, for the immunotherapy agents: anti-CTLA-4 (ipilimumab) or anti-PD-1 (pembrolizumab, nivolumab) monoclonal antibodies which result in activation and proliferation of immune effector cells [25,26]. Each of the indicated therapies has limited efficacy and secondary resistance develops over time. Ipilimumab treatment was referred to as effective in only 22% and PD-1 inhibitors up to 40–45% of patients with melanoma after 5–10 years of therapy [27].

Due to the growing number of available types of therapeutic agents, as well as the approval of combination therapies, there is an understandable need for establishing the biomarkers of response to particular types of treatment to stratify the patients to the most promising therapy for them, based on scientific evidence. Various potential biomarkers of response to immunotherapy are currently in debate. In our previous paper, we comprehensively review a number of them presented from the tumour or host perspective and their correlation with immunotherapy effectiveness [28].

In this study, we revealed the potential role of one of them: MDSCs including the subtypes of these immunosuppressive cells as a biomarker of response to anti-PD-1 therapy in advanced melanoma patients. The long-term PFS analysis is presented in the context of frequencies of MDSCs. Moreover, we analysed the changes in cell frequencies during the treatment and correlated our results with the serum level of lactate dehydrogenase (LDH), which has been previously related to the occurrence of response to immunotherapy [29,30,31].

## 2. Materials and Methods

### 2.1. Study Design, Samples and Ethical Statements

In this retrospective study, we analysed the changes in the populations of MDSC in advanced melanoma patients before and during anti-PD-a therapy. Patients who were qualified for treatment with anti-PD-1 antibodies (pembrolizumab or nivolumab) at the Department of Medical and Experimental Oncology of Poznan University of Medical Sciences were invited to participate in the study without additional criteria. The dosage for patients treated with nivolumab was 480 mg infused in cycles every 4 weeks and for patients treated with pembrolizumab, it was 400 mg in cycles every 6 weeks. Samples of peripheral blood were obtained from patients with metastatic melanoma (n = 46) at two time points: at the time of initiation of therapy (baseline, BL) and in the 3 months of therapy (T3). T3 patients treated with nivolumab received 3 cycles of therapy and pembrolizumab, 2 cycles. Patients with infectious diseases were excluded from this study. The evaluation of response to anti-PD-1 treatment was assessed using Response Evaluation Criteria in Solid Tumors (RECIST) 1.1. in the third month of therapy. Patients with a partial response to the treatment (PR) and with stable disease (SD) were considered as responders (R), and patients with progressive disease (PD) as non-responders (NR) to anti-PD-1 antibodies in this study. Blood samples from healthy donors (HD) (n = 9) were obtained only once. Peripheral blood was obtained following written consent of participation. The study was approved by the Bioethics Committee of Poznan University of Medical Sciences (study number 402/18). Details on patients’ characteristics are summarised in Table 1.

### 2.2. PBMC Separation and Cryopreservation

Blood was collected in vacutainer tubes with an anticoagulant (heparin) (#367874, BD Biosciences). Peripheral mononuclear cells (PBMC) were isolated within 3 h of blood donation by standard gradient centrifugation in a Histopaque 1077 (#SD10771B, Sigma-Aldrich, St. Louis, MO, USA). Cells were cryopreserved using the CTL-Cryo ™ ABC Media Kit (#CTLC-ABC, CTL, Cleveland, OH, USA), as described previously [58] and stored in the vapour phase of liquid nitrogen until testing. Samples were frozen for a maximum duration of three years. Thawed and washed twice in phosphate-buffered saline (PBS) solution, the PBMCs pellet was used for further analyses.

### 2.3. Flow Cytometry for Myeloid-Derived Suppressor Cells and Subsets

Cell immunophenotyping was performed by flow cytometry using the following monoclonal antibodies (mAbs): CD33-PE-Cy™7 (#333946, BD Biosciences, Franklin Lakes, NJ, USA), CD11b-APC (#550019, BD Biosciences, Franklin Lakes, NJ, USA), CD45-APC-Cy™7 (#557833, BD Biosciences, Franklin Lakes, NJ, USA) CD66b-FITC (#555724, BD Biosciences, Franklin Lakes, NJ, USA), CD14-PerCP-Cy™5.5 (#562692, BD Biosciences, Franklin Lakes, NJ, USA), HLA-DR-PE (#555812, BD Biosciences, Franklin Lakes, NJ, USA). Antibodies were added to the cytometric tubes containing PBMC pellets resuspended in 100 µL of PBS solution. Samples were mixed gently by vortex and incubated for 15 min at room temperature and protected from light. After this time, 500 µL of cell lysis solution (#349202, BD Biosciences, Franklin Lakes, NJ, USA) was added to the test tubes and the tubes were incubated for the next 10 min, followed by washing with 2 mL of PBS solution. Samples were centrifuged at 1500 rpm for 5 min at room temperature. The supernatants were discarded. This step was repeated twice. The cell pellets were resuspended in 200 µL of PBS solution. The samples were then acquired using a FACS Aria flow cytometer (BD Biosciences, Franklin Lakes, NJ, USA). The obtained results were analysed with the FACS Diva software v. 6.1.3 (Becton Dickinson, Franklin Lakes, NJ, USA), integrated with the cytometer. For each examined antibody, the percentage of positive cells and mean fluorescence intensity (MFI) were determined.

### 2.4. Progression-Free Survival Analysis and Definition of Cut-Offs

Patient progression-free survival (PFS) was measured starting the day of the first administration of anti-PD-1 until the occurrence of progression. As was mentioned before, the evaluation of response to anti-PD-1 treatment was assessed in the third month of therapy. For this reason, the length of PFS for responders exceeds 3 months, while for non-responders it is about 3 months maximum. Since the results of cytometric analyses showed a wide range of results for the group of responders, we determined cut-offs of those parameters and divided the responders into populations above or below the distinguished level, then compared their PFS. Cut-offs were determined to compare responders’ PFS in the context of cell levels. The cut-off values were the median cell levels observed in R (MDSC total, GrMDSC and MoMDSC). However, ImMC distribution in R was different, due to the low level of these cells in both analysed groups, therefore the cut-off value for these cells was determined as a median cell level observed in NR group. PFS analysis was performed by using the Kaplan–Meier method and GraphPad Prism Software Prism 9.0 (La Jolla, CA, USA). Survival curves were compared using the log-rank (Mantel–Cox) test.

### 2.5. Statistical Analysis

Statistical analyses were performed with GraphPad Prism Software Prism 9.0 (La Jolla, CA, USA) including parametric and non-parametric tests (Kruskal–Wallis test and *t*-Student test), *p* values ≤ 0.05 were considered statistically significant.

## 3. Results

### 3.1. Patients Characteristics

The total number of advanced melanoma patients enrolled in the study was 46. Patients qualified for treatment with anti-PD-1 antibodies (pembrolizumab or nivolumab) at the Department of Medical and Experimental Oncology of Poznan University of Medical Sciences consented to participate in this study were enrolled without additional criteria. The mean age of melanoma participants was 63 ± 10.5 years, the youngest patient was 32 and the oldest 92 years old. Nineteen (41.3%) of the enrolled participants were female and twenty-seven (58.7%) were male. Over 95% of patients were diagnosed at the IV stage and only two of them (4.3%) at the III stage of disease. The type of therapy was decided by the patient’s oncologist: 23 (50%) of the patients were treated with nivolumab and the other 23 received pembrolizumab. The length of the therapy cycle was various, nivolumab was given every 4 weeks and pembrolizumab was every 3 weeks. For 38 patients (82.6%) it was the first and for 8 (17.4%) the second line of treatment. This study enrolled nine healthy volunteers, four (44.4%) male and five (55.6%) female. The mean age of them was 52 ± 9.4 years, the youngest was 32 and the oldest 67 years old. Patients’ and healthy volunteers’ characteristics are summarized in Table 1.

### 3.2. Overall Clinical Response in Melanoma Patients Treated with Anti-PD-1 Immunotherapy

Clinical response was evaluated after three cycles of anti-PD-1 therapy. The overall clinical response for both types of anti-PD-1 therapy (nivolumab and pembrolizumab) (n = 46) was as follows: 19 (41.3%) patients had a partial response (PR), 8 (17.4%) had stable disease (SD) and 19 (41.3%) a progressive disease (PD). In this study, we defined responders to anti-PD-1 therapy as patients with a PR and SD (n = 27), and non-responders as patients with PD (n = 19). More than half (58.7%) of patients experienced a progression of melanoma in 1 year. Then, we analysed the overall clinical response based on the type of anti-PD-1 therapy. The same number of patients in this study was treated with nivolumab (n = 23, 50%) or pembrolizumab (n = 23, 50%). Eight (34.8%) of the patients treated with nivolumab had a PR, five (21.7%) SD and ten (43.5%) had a PD. Over half (52.2%) of the patients treated with nivolumab experienced a progression within 1 year. Eleven (47.8%) of the patients treated with pembrolizumab had a PR, three (13.0%) SD and nine (39.1%) a PD to the therapy. We observed that patients treated with pembrolizumab were more likely to experience a progression within 1 year compared to nivolumab (65.2% and 56.5%, respectively). Patients with the clinical benefit of anti-PD-1 treatment statistically later experienced disease progression compared to non-responders. The median progression-free survival (PFS) for responders was 22 ± 9.6 and for non-responders 2.3 ± 0.6 months (*p* < 0.0001).

### 3.3. Levels of Circulating ImMC, GrMDSC, MoMDSC and MDSC Total Cells before the First Drug Administration

**Figure 1:**
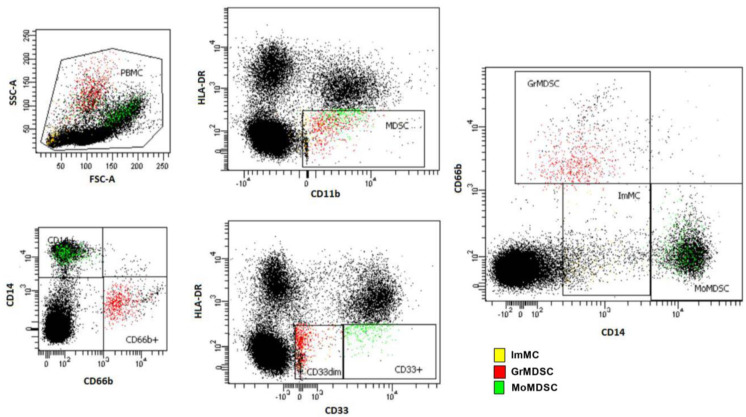
Representative flow cytometry gating for MDSCs as CD11b^+^/HLA-DR^−^/low/CD33^+^, immature MC (ImMC): CD14^−^/low/CD33low/CD11b^+^/HLA-DR^−^/low, monocytic MDSC (MoMDSC): CD14^+^/CD33high/CD11b^+^/HLA-DR^−^/low and granulocytic MDSC (GrMDSC): CD66b^+^/CD33dim/CD11b^+^/HLA-DR^−^/low.

**Figure 2:**
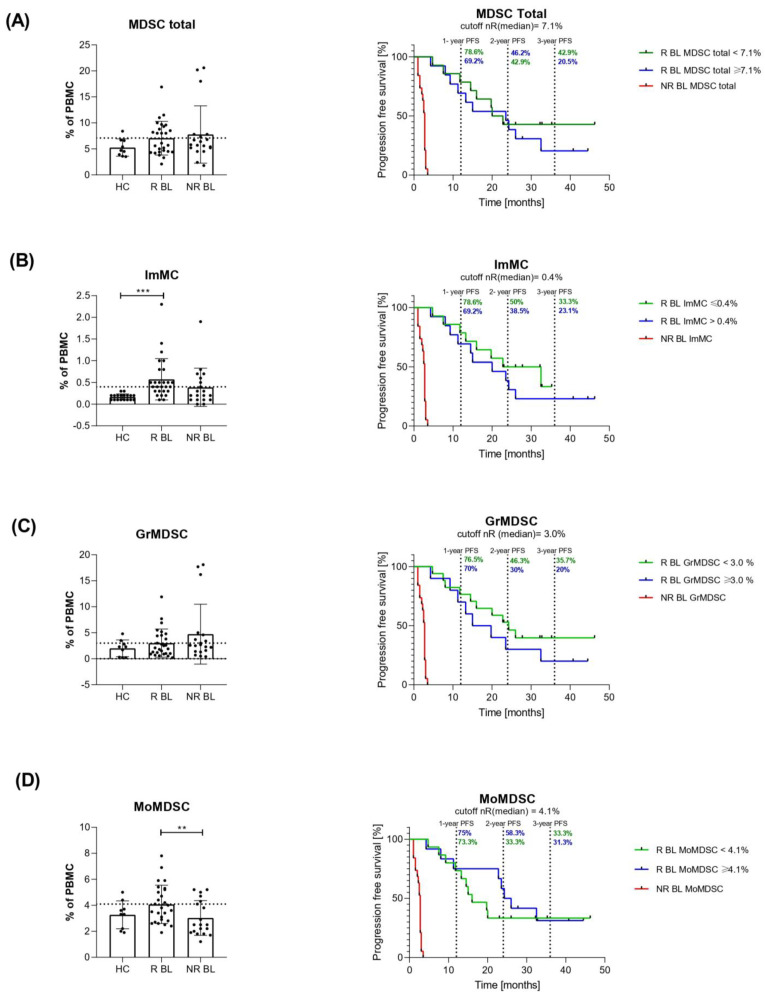
Myeloid suppressor cell (MDSC) levels prior to immunotherapy. Levels of circulating MDSC total, ImMC, GrMDSC and MoMDSC were evaluated as % of PBMC and analysed using Kruskal-Wallis and t-Student tests. Occurrence of the response to anti-PD-1 therapy was evaluated in the third month of treatment. Responders group (R) included patients with PR and SD, non-responders (NR)-PD. For progression-free survival (PFS) analysis of responders and non-responders to immune checkpoint inhibitor (ICI) therapy (log-rank (Mantel–Cox) test was performed. (**A**) Levels of total MDSC grouped by patients’ response to anti-PD-1 therapy and the healthy control. The dashed line marks the median level for R (7.1%). Log-rank (Mantel–Cox) test, PFS analysis *p* (MDSC total, NR BL vs. R BL < 7.1%) < 0.0001, *p* (MDSC total, NR BL vs. R BL ≥ 7.1%) < 0.0001. (**B**) Levels of immature monocytic cells (ImMC), *p* (ImMC, HC vs. R BL) = 0.0019. The dashed line marks the median level for NR (0.4%). Log-rank (Mantel–Cox) test, PFS analysis *p* (ImMC, NR BL vs. R BL ≤ 0.4%) < 0.0001, *p* (ImMC, NR BL vs. R BL > 0.4%) < 0.0001, (**C**) granulocytic MDSC (GrMDSC). The dashed line marks the median level for R (3.0%). Log-rank (Mantel–Cox) test, PFS analysis *p* (GrMDSC, NR BL vs. R BL < 3.0%) < 0.0001, *p* (GrMDSC, NR BL vs. R BL ≥ 3.0%) < 0.0001 and (**D**) monocytic MDSC (MoMDSC), *p* (MoMDSC, R vs. NR) = 0.0333. The dashed line marks the median level for NR (4.1%). Log-rank (Mantel–Cox) test, PFS analysis *p* (MoMDSC, NR BL vs. R BL < 4.1%) < 0.0001, *p* (MoMDSC, NR BL vs. R BL ≥ 4.1%) < 0.0001. Data are shown as a mean ± SD, ** *p* < 0.02, *** *p* < 0.005. PR, partial response; SD, stable disease; PD, progressive disease.

We analysed the level of circulating myeloid-derived suppressor cells (MDSC) and their subpopulations in total peripheral mononuclear cells (PBMCs) of melanoma patients before anti-PD-1 therapy (baseline, BL). The gating strategy is presented in Figure 1. MDSC were defined as CD11b^+^/HLA-DR^−^/low/CD33^+^. Moreover, MDSC subsets were determined as immature MC (ImMC): CD14^−^/low/CD33low/CD11b^+^/HLA-DR^−^/low, monocytic MDSC (MoMDSC): CD14^+^/CD33high/CD11b^+^/HLA-DR^−^/low and granulocytic MDSC (GrMDSC): CD66b^+^/CD33dim/CD11b^+^/ HLA-DR^−^/low. The total MDSC for healthy controls (HC) comprised 5.3 ± 1.4% of all PBMC and for responders (R) and non-responders (NR) to anti-PD-1 therapy before the treatment (baseline, BL), was 7.1 ± 2.6% and 7.8 ± 3.7%, respectively (Figure 2). The levels of ImMC in HC were 0.2 ± 0.1%, the NR group was 0.4 ± 0.3% and the R was 0.6 ± 0.3% which was three-fold higher compared to HC (*p* = 0.0019). We observed no significant differences in GrMDSC level between analysed groups of patients, though melanoma patients had 3.0 ± 2.0% and 4.7 ± 4.0% (R and NR, respectively), compared to 2.0 ± 1.3% for HC. In contrast, the level of MoMDSC was significantly higher in R (4.1 ± 1.2%) compared to NR patients (3.0 ± 1.2%) (*p* = 0.0333). Individual dot plots of the data are presented in Figure 2.

### 3.4. Progression-Free Survival Analysis of Patients Depending on Myeloid-Derived Suppressor Cells Rate

Due to the observation of relatively similar myeloid-derived suppressor cell distribution between groups of patients, we determined the cut-offs to differentiate responders with the upper level of analysed cells above the cut-off, and the lower level below the cut-off. We hypothesised a shortened PFS in responders with cell levels in the range of non-responders. We compared PFS between those responder groups and non-responders. The follow-up span allowed us to point out the 1-, 2- and 3-year PFS of responders, although most non-responders’ PFS did not reach 3 months due to cases of death and patients’ response determination at the 3rd month of therapy. Responders to anti-PD- with MDSC total below 7.1% had very similar 1- and 2-year PFS to patients with MDSC total above the cut-off (78.6 vs. 69.2% and 42.9 vs. 46.2%, respectively), however, the 3-year PFS for this group was 42.9 vs. 20.5% for responders with MDSC total below and above the cut-off, respectively. The lower level of ImMC (below the cut-off of 0.4%) in responders was related to longer PFS compared to ImMC above the cut-off: 78.6 vs. 69.2%, 50 vs. 38.5% and 33.3 vs. 23.1% for 1-, 2- and 3-year PFS, respectively. Additionally, the lower level of GrMDSC favoured the longer PFS for responders to anti-PD-1 therapy. Patients with baseline GrMDSC below and above the cut-off (3.0%) reached 76.5 vs. 70% 1-year PFS, 46.3 vs. 30% 2-year PFS and 35.7 vs. 20%, respectively. Analysis of 1- and 3-year PFS in responders did not reveal differences greater than 2% between responders below and above 4.1% of MoMDSC. Interestingly, the higher level of MoMDSC favoured the longer 2-year PFS than below the cut-off (58.3 vs. 33.3%) (Figure 2).

### 3.5. Levels of ImMC, GrMDSC, MoMDSC and MDSC Total Cells in HC Depending on the Age

**Figure 3:**
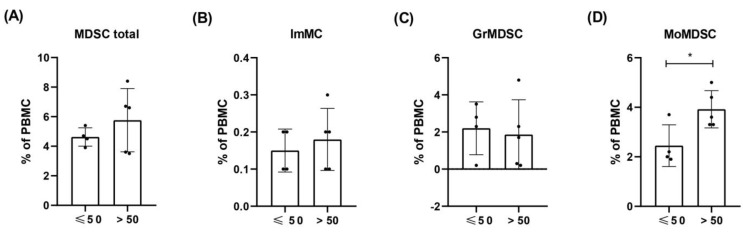
Levels of circula ting ImMC, GrMDSC, MoMDSC and MDSC total cells in HC depending on the age. Healthy volunteers were divided into two groups based on their age: ≤50 (the mean age 42 *±* 7.75 years) and >50 (the mean age 63 *±* 6.16 years) and compared cell levels between them. MDSCs levels were evaluated as % of PBMC. Levels of (**A**) total MDSC, (**B**) immature monocytic cells (ImMC), (**C**) granulocytic MDSC (GrMDSC) and (**D**) monocytic MDSC (MoMDSC). Data are shown as a mean ± SD, * *p* ≤ 0.05.

For comparative analyses of cell levels in healthy volunteers and melanoma patients, we use the average values for all nine healthy volunteers, signed as healthy control (HC). However, due to the age variation of the volunteers and the lower average age of them compared to the patients, we conducted an analysis in which we divided HC into two groups: under the age of 50 (the mean age 42 ± 7.75 years) and over 50 (the mean age 63 ± 6.16 years) and compared cell levels between them. We observed higher levels of MoMDSCs in the older (3.92 ± 0.62%) compared to the younger healthy donors (2.45 ± 0.63) (*p =* 0.0279). The levels of other MDSCs were at a similar level between the analysed groups (Figure 3).

### 3.6. Levels of Circulating ImMC, GrMDSC, MoMDSC and MDSC Total Cells Following Immunotherapy

To determine how the levels of cells and subpopulations changed during therapy with anti-PD-1, we analysed PBMCs of melanoma patients at two time points: before the treatment (BL) and in the third month of therapy (T3). Responders to anti-PD-1 in the third month of therapy had higher levels of MDSC total cells compared to HC, 7.3 ± 2.2% and 5.3 ± 1.4%, respectively (*p* = 0.0433).

**Figure 4:**
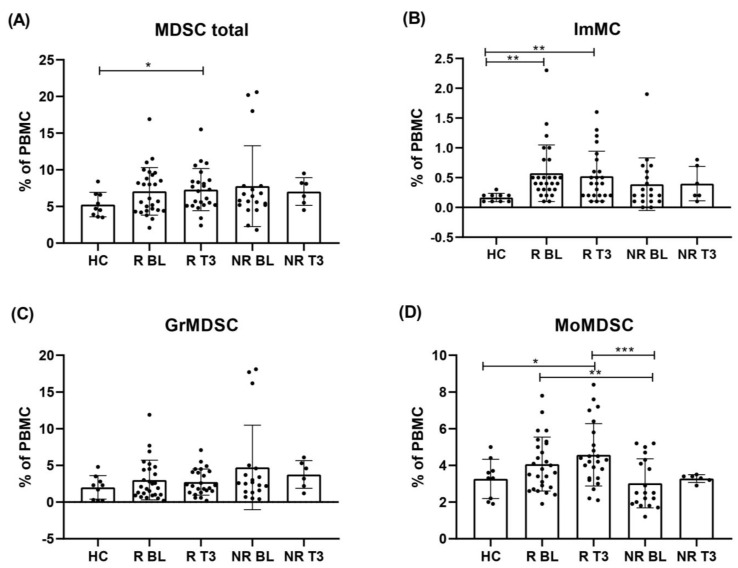
Levels of circulating MDSC total cells, ImMC, GrMDSC and MoMDSC following immunotherapy. Patients’ MDSC levels were evaluated as % of PBMC and presented in the context of clinical benefit in two time points: baseline (BL) and third month of anti-PD-1 therapy (T3). Occurring response to therapy was measured in the third month of treatment. Responders group (R) included patients with PR and SD, non-responders (NR)- PD. (**A**) Levels of total MDSC grouped by patients’ response to anti-PD-1 therapy. Levels of (**B**) immature monocytic cells (ImMC), (**C**) granulocytic MDSC (GrMDSC) and (**D**) monocytic MDSC (MoMDSC). Data are shown as a mean ± SD, * *p* ≤ 0.05, ** *p* < 0.02, *** *p* < 0.005. PR, partial response; SD, stable disease; PD, progressive disease.

The level of ImMC in responders in T3 was significantly higher compared to HC (0.5 ± 0.3 and 0.2 ± 0.1, respectively) (*p* = 0.0162). In the non-responders’ group, we did not notice changes in this particular subpopulation of cells after 3 months of treatment. The level of MoMDSC cells in the third month of therapy in R was 39% higher than observed in HC (4.6 ± 1.3% and 3.3 ± 0.8%, respectively) (*p* = 0.0394). Interestingly, the level of MoMDSCs in the responders’ group in T3 was 35% higher than BL in non-responders (*p* = 0.0022). During anti-PD-1 therapy, levels of GrMDSC decreased in melanoma patients compared to BL regardless of the occurrence of response to the treatment, nevertheless, these differences were not statistically significant (Figure 4).

### 3.7. Lactate Dehydrogenase Level in Responders and Non-Responders to Checkpoint Therapy

**Figure 5:**
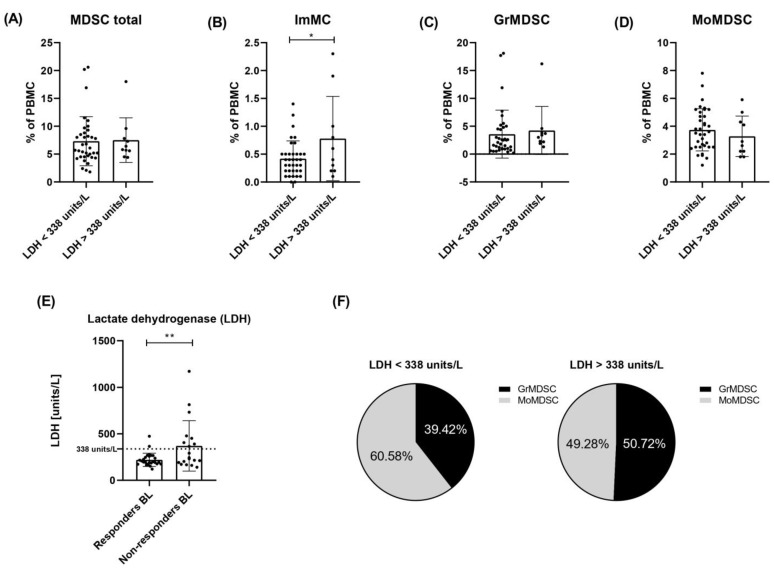
Levels of circulating MDSC total cells, ImMC, GrMDSC and MoMDSC in the context of serum lactate dehydrogenase level (LDH). Patients’ MDSC levels were evaluated as % of PBMC. Patients were divided into two groups based on baseline LDH serum level: below 338 units/L (n = 36) and above 338 units/L (1.5 times elevated normal LDH serum level) (n = 10). The groups included both responders and non-responders to anti-PD-1 therapy. Levels of (**A**) total MDSC, (**B**) immature monocytic cells (ImMC), (**C**) granulocytic MDSC (GrMDSC) and (**D**) monocytic MDSC (MoMDSC). Correlation between lactate dehydrogenase (LDH) serum level and response to therapy. (**E**) Mean LDH concentration measured before first administration observed in non-responders was 1.68-fold higher compared to responders to anti-PD-1 therapy (370.58 ± 194.94 and 220.85 ± 50.26 units/L), *p* = 0.0086. Percentage distribution of GrMDSCs and MoMDSCs in total MDSCs in patients with LDH level < or > 338 units/L (**F**). Data are shown as a mean ± SD, * *p* ≤ 0.05, ** *p* < 0.02.

Patients’ serum LDH levels were measured before the first administration of anti-PD-1 therapy. We observed a 1.68-fold higher mean LDH concentration in a group of non-responders compared to responders to anti-PD-1 therapy (370.58 ± 194.94 and 220.85 ± 50.26 units/L, respectively, *p* = 0.0086). Eight of nineteen (42.11%) non-responders had LDH serum levels above 338 units/L (>1.5 × upper limit of normal concentration), and only two of twenty-seven (7.41%) responders exceeded that upper level (Figure 5E).

### 3.8. Analysis of Myeloid-Derived Suppressor Cells in Relation to Elevated LDH Level

We re-analysed the obtained results in the context of LDH serum level (not as previously, the occurrence of response to therapy). We compared the levels of circulating MDSCs, ImMCs, MoMDSCs and GrMDSCs between two groups of patients with LDH levels below 338 (n = 36) and above 338 units/L (n = 10). The cut-off value corresponded with a 1.5-fold exceeded norm of this parameter (225 units/L). Both groups included responders and non-responders to anti-PD-1 therapy. We observed no significant differences in the level of circulating MDSCs, MoMDSCs or GrMDSC cells between analysed groups of melanoma patients. However, the level of ImMCs was higher in the group of patients with LDH level > 338 units/L (0.78 ± 0.58%) compared to those with <338 units/L (0.42 ± 0.23%) (*p* = 0.0285) (Figure 5). Then, we analysed the percentage distribution of GrMDSCs and MoMDSCs in total MDSCs. Patients with LDH level below 338 units/L MDSCs consisted of 39.42 ± 19.07% GrMDSCs and 60.58 ± 18.96% MoMDSCs (*p* = 0.0002), patients with LDH above 338 units/L MDSCs: 49.28 ± 14.21% GrMDSC and 50.72 ± 14.25% MoMDSC (Figure 5F).

## 4. Discussion

In the present study, we evaluated the potential role of myeloid-derived suppressor cells as an immune signature of response to anti-PD-1 therapy in melanoma patients. We performed the analysis not only of the MDSCs but also included three subtypes of these cells. It allowed us to distinguish the relationship between them, indicate the role of each of them in developing a response to immunotherapy and verify the effect of the treatment on cell frequencies.

The potential role of MDSCs as a biomarker of response to cancer immunotherapy stays in focus for many reasons. First of all, these inhibitory immune cells have a well-characterized role in modulating the response to cancer immunotherapies. The results of many studies, not only in melanoma, indicate their potential prognostic value. Moreover, the determination of blood parameters is convenient due to easy access and the ability to monitor multiple times during the treatment.

In the current study, we observed a higher baseline MoMDSC level in responders to anti-PD-1 therapy, compared to non-responders. In a previous study, melanoma patients treated with anti-CTLA showed a similar frequency of this subset for both groups of patients, moreover, patients with lower MoMDSCs levels were more likely to benefit from this immunotherapy [32]. These observations might result from the differences between types of ICI and require further investigation. The baseline MDSCs frequencies were similar for all melanoma patients, however, non-responders to anti-PD-1 were the group with a higher diversity of levels of these particular cells. Analysis of PFS showed a higher 3-year % for responders with MDSC levels above 7.1%. According to this, a low baseline MDSC level was associated with the highest probability of long-term survival in melanoma patients treated with ipilimumab [32]. Higher CD66^+^/CD33dim cells frequency was observed for non-responders, and even though this was not a significant value, the same tendency was already reported for melanoma patients treated with various immunotherapies [33]. Lower GrMDSC level was related to a higher 3-year PFS for responders to anti-PD-1. Krebs et al. reported higher 2-year survival rate in OS analysis for non-responders to immunotherapy with a GrMDSCs < 0.5% of PBMCs [33].

Responders to anti-PD-1 had higher ImMCs level compared to non-responders, but we also observed that higher baseline frequencies of these cells were preferable in 3-years PFS analysis. Melanoma patients had a higher MDSCs frequency compared to healthy donors, but this difference is the most evident for immature monocytic cells. It was already reported that melanoma patients have higher MDSC levels than healthy controls, what is more, the frequency of circulating MDSC correlates with the tumour burden and disease stage. So, in patients with more advanced melanoma, but also NSCLC, pancreatic or bladder cancer higher frequencies of MDSC were detected in the peripheral. The level of these immune cells could be considered an indicator of other unfavourable parameters in cancers [34,35,36,37].

However, we should keep in mind that circular MDSCs level may not reflect the exact tumour microenvironment (TME) infiltration status and that GrMDSCs and MoMDSCs in the TME are more suppressive than in peripheral lymphoid organs or peripheral blood. After migration to the tumour, conditions such as hypoxia stimulate the expression and secretion of various immunosuppressive molecules by MDSCs, resulting in a highly suppressive environment in tumours and preventing the rejection of tumours via immune-mediated mechanisms [38]. It was already observed in a mouse model study, that splenic GrMDSCs strongly suppress CD4^+^ T-cell proliferation while the suppressive effect on CD8^+^ T cells is less pronounced compared to tumour GrMDSCs. Tumour-derived MoMDSCs produce more NO2^−^ and both tumour-derived subsets have enhanced arginase activity [39].

Our results present no significant difference in MDSCs frequencies during the treatment regardless of the occurrence of response to the treatment. Similar findings were presented by Meyer et al. in a study of melanoma patients treated with ipilimumab [40]. However, changes in frequencies of immunosuppressive cells following the administration of the immune checkpoint therapy were observed in other studies. Sun et al. in a comprehensive study on 128 advanced melanoma patients treated with immunotherapy described the significant increase in MDSCs in the second and third cycle of anti-PD-1 administration compared to baseline, however, it was not differentiated in the type of response to the treatment [41]. For monitoring the dynamics of changes in peripheral MDSCs frequencies it is crucial to determine blood collection time points. Comparing the results from studies with different time points and other diversities, such as the stage of disease or the type of therapy can be very misleading.

During a lifetime, the number of circulating MDSCs increases, and higher levels are observed in elderly people especially older than 65 years [42,43]. This is due to chronic inflammation and a process of immunosenescence in which immune dysfunction develops during a life span, the resulting occurrence of autoimmune diseases or malignant tumours [44,45,46]. In our study, the mean age of healthy volunteers, an informative control providing physiological MDSC levels, was on average 10 years younger than the mean age of patients in this study. This is one of the limitations of the study that we are aware of. Therefore, based on the previous studies suggesting that age-related increase in myelopoiesis can enhance the production of myeloid cells, including MDSCs [46], we analysed the distribution of MDSCs in smaller groups of healthy participants based on their age: below or above 50 years of age. In the group of older healthy volunteers, we observed higher levels of MDSCs and especially MoMDSCs. This result is only partially consistent with previous studies, in which an increase in the level of GrMDSC was observed with age. This may be due to the mean age differences of the studied groups. Verschoor et al. recruited much older participants (over 61 to 99 years old) [42], however, our results indicate some differences observed in younger groups of healthy participants which can also be informative. Our observations suggest that the age of participants (both, the control group and patients) should be as similar as possible.

LDH is a well-known biomarker for poor outcomes in metastatic melanoma patients. Elevated LDH level is a negative prognostic factor regardless of the type of treatment and has been included in the current version of the AJCC staging system [47]. Previous results have already confirmed this phenomenon, some of them pointed to the exact concentration of elevated LDH levels as a cut-off in the analysis of OS in melanoma patients [30,32,33,48]. A strong correlation between low LDH levels and the occurrence of response to anti-PD-1 therapy was observed in this study. LDH serum level above 1.5 × upper limit of normal concentration correlated with no response to treatment. However, as we present, non-responders to anti-PD-1 had a wide range of LDH concentrations and only less than half of them had LDH levels high above the normal concentration. What is interesting, some of the patients whose LDH concentration exceeded 1.5-fold of the normal level still benefited from anti-PD-1 therapy. Nonetheless, according to previous studies, LDH serum levels before the first administration of anti-PD-1 in the group of responders’ therapy were lower compared to non-responders [33,49,50].

In this study, we did not observe different MDSC frequencies in PBMCs of patients with elevated LDH (above 1.5 × upper limit of normal concentration), although elevated levels of LDH correlated with poor response to anti-PD-1 therapy. These results are consistent with the results for melanoma patients treated with ipilimumab. Meyer et al. considered possible altered recruitment of MDSCs in the TME of patients with higher serum LDH levels that may remain undetectable in the peripheral blood [40]. We found that an LDH level above the cut-off value was associated with elevated ImMC baseline levels. What is interesting, we did not observe high differences in the level of these particular cells in comparison between responders and non-responders. This may suggest a direction for further research in which this parameter would be investigated as a prognostic factor only in the context of the LDH level.

Next, we observed a changed distribution of MDSCs populations (GrMDSCs and MoMDSCs) between groups with LDH levels below or above the cut-off level, regardless of the occurrence of response to therapy. Patients with lower LDH level had higher participation of MoMDSCs than GrMDSCs in the MDSC population, while elevated LDH level was related to a similar ratio of both subpopulations. Elevated levels of LDH as well as GrMDSCs have previously been indicated as negative prognostic factors in response to treatment and OS [33,41]. As was presented before, melanoma among other solid tumours such as prostate cancer or multiple myeloma presents an expansion of MoMDSC over GrMDSCs in the peripheral, and this is in agreement with our results for the vast majority of our patients [51].

Technical aspects of the study are also worth mentioning. The classification, method of phenotyping and definition of MDSC subpopulations is in debate.

Characterization of MDSCs in this study was performed by common MDSCs surface markers: CD11b^+^, HLA-DR-/low and CD33^+^. MDSCs subsets were distinguished by specific markers: CD14^+^ and CD33high for monocytic MDSCs, CD66b^+^ and CD33dim for granulocytic MDSCs and CD14^−^/low and CD33low for ImMCs. The protocols used in studies differ, because of the analytic limitations of the cytometer used for work or gating strategies. For example, CD15^+^ is a popular marker of GrMDSCs, however, CD66^+^ does not differ in usefulness in this manner [41,52].

The various protocols are currently in use; however, some certain elements are indicated as affecting the final result. Differences occur at many levels of the test, starting from the type of anticoagulant in the test tubes (heparin, EDTA, sodium citrate), through the method of performing the assay (whole blood or separated PBMC cells, type of reagents for PBMC cell separation), ending with the determination of selected surface markers and gating strategies of marked cells. The multicentre study on the influence of blood collection tube type and cell separation medium shows that certain conditions may increase the concentration of polymorphonuclear MDSCs [53].

In recent years, there has been an increase in interest in MDSC due to its suppressive role in tumour progression. Due to the exponentially growing number of studies in this manner, Bronte et al. presented the recommendation of conditions for the analysis of MDSC [54]. Although the flow cytometric analysis of cryopreserved blood cells is discouraged [40,55], it was practised in many studies [32,35,36,56,57]. In addition, for us, it was not possible to perform analysis on fresh blood samples. To minimize the potential negative effect of cell cryopreservation, we carried out freezing using reagents of the manufacturer that ensures the preservation of cell properties and their surface markers after thawing the sample. Moreover, we have had a positive experience conducting analyses hereby in the past [58,59].

In conclusion, searching for one particular blood-related parameter as a biomarker of response to melanoma immunotherapy could be ineffective and inconclusive. Especially when the differences in the level of the analysed parameters amount to a few percentages, their biological significance most likely results from the diversity of other parameters (biomarkers), which should be considered together. Due to it, Martens et al. proposed a combined analysis of the baseline frequencies of MDSCs, Tregs, LDH and routine blood counts with clinical characteristics as a tool for predicting outcomes following ipilimumab therapy for advanced melanoma patients [32]. Although our study was retrospective and involved a relatively small number of patients and healthy controls, it suggests that MDSCs frequencies may not directly correlate with the outcome of anti-PD-1 treatment as was suggested before [60]. Our results present new insight into monocytic MDSC as a possible biomarker, however more detailed and mechanistic studies are required to clarify this issue.

## Figures and Tables

**Table 1:** Demographic and clinical data for advanced melanoma patients included in the study and healthy volunteers.

Patients Characteristics	Responders (n = 27)	Non-Responders (n = 19)	Healthy Control (n = 9)
Age (years)			
Mean	61.19	66.16	52
Median	63	66	51
Min, Max	32, 85	38, 92	32, 67
SD	11.44	9.01	9.43
Gender, n (%)			
Male	15 (55.56)	12 (63.16)	4 (44.44)
Female	12 (44.44)	7 (36.84)	5 (55.56)
Stage at diagnosis, n (%)			
III c	2 (7.41)	0 (0.00)	
IV total	25 (92.59)	19 (100.00)	
IV M1a	11 (40.74)	2 (10.53)	
IV M1b	4 (14.81)	3 (15.79)	
IV M1c	8 (29.63)	10 (52.63)	
IV M1d	2 (7.41)	4 (21.05)	
BRAF mutation status, n (%)			
BRAF −	16 (59.26)	7 (36.84)	
BRAF +	11 (40.74)	12 (63.16)	
Immunotherapy			
Nivolumab	13 (48.15)	10 (52.63)	
Pembrolizumab	14 (51.85)	9 (47.37)	
The line of treatment, n (%)			
I	22 (81.48)	16 (84.21)	
II	5 (18.52)	3 (15.79)	
Best overall response, n (%)			
Partial response (PR)	19 (70.37)	0 (0.00)	
Stable disease (SD)	8 (29.63)	0 (0.00)	
Progressive disease (PD)	0 (0.00)	19 (100.00)	
Progression within 1 year, n (%)			
Yes	8 (29.63)	19 (100.00)	
No	19 (70.37)	0 (0.00)	
Progression-free survival (months)			
Median	21.97	2.33	
SD	9.66	0.64	

## Data Availability

The data presented in this study are available on request from the corresponding author upon reasonable request.
